# Targeting a Designer TIMP-1 to the Cell Surface for Effective MT1-MMP Inhibition: A Potential Role for the Prion Protein in Renal Carcinoma Therapy

**DOI:** 10.3390/molecules24020255

**Published:** 2019-01-11

**Authors:** Bingjie Jiang, Jian Liu, Meng Huee Lee

**Affiliations:** Department of Biological Sciences, Xian Jiaotong Liverpool University, 111 Ren Ai Road, Suzhou 215123, China; bingjie518@126.com or bingjie.jiang@xjtlu.edu.cn (B.J.); jian.liu@xjtlu.edu.cn (J.L.)

**Keywords:** MT1-MMP, TIMP, renal carcinoma, cancer therapy, prion, GPI anchor, protein engineering

## Abstract

Renal carcinoma cells express Membrane Type 1-Matrix Metalloproteinase (MT1-MMP, MMP-14) to degrade extracellular matrix components and a range of bioactive molecules to allow metastasis and cell proliferation. The activity of MT1-MMP is modulated by the endogenous inhibitors, Tissue Inhibitor of Metalloproteinases (TIMPs). In this study, we describe a novel strategy that would enable a “designer” TIMP-1 tailored specifically for MT1-MMP inhibition (V4A/P6V/T98L; *K*_i_^app^ 1.66 nM) to be targeted to the plasma membrane for more effective MT1-MMP inhibition. To achieve this, we fuse the designer TIMP-1 to the glycosyl-phosphatidyl inositol (GPI) anchor of the prion protein to create a membrane-tethered, high-affinity TIMP variant named “T1^Pr αMT1^” that is predominantly located on the cell surface and co-localised with MT1-MMP. Confocal microscopy shows that T1^Pr αMT1^ is found throughout the cell surface in particular the membrane ruffles where MT1-MMP is most abundant. Expression of T1^Pr αMT1^ brings about a complete abrogation of the gelatinolytic activity of cellular MT1-MMP in HT1080 fibrosarcoma cells whilst in renal carcinoma cells CaKi-1, the GPI-TIMP causes a disruption in MMP-mediated proteolysis of ECM components such as fibronectin, collagen I and laminin that consequently triggers a downstream senescence response. Moreover, the transduced cells also suffer from an impairment in proliferation and survival in vitro as well as in NOD/SCID mouse xenograft. Taken together, our findings demonstrate that the GPI anchor of prion could be exploited as a targeting device in TIMP engineering for MT1-MMP inhibition with a potential in renal carcinoma therapy.

## 1. Introduction

Membrane Type-1 Matrix Metalloproteinase (MT1-MMP, MMP-14) is a prominent member of the zinc-dependent Matrix Metalloproteinase (MMP) family best known for its involvement in the modulation of the extracellular environment and cellular processes such as cell invasion and proliferation [1,2,3]. Among the large repertoire of extracellular matrix (ECM) and basement membrane components cleaved by MT1-MMP are the adhesion molecules and fibrillar proteins fibronectin, vitronectin, laminins, collagens I, II and III [4,5] as well as key signalling molecules implicated in cancer dissemination such as CD44, syndecan-1, RANKL and MUC1 [6,7,8,9]. Like the other members of the MMP family, MT1-MMP is a multi-domain proteinase consisting of a pro-domain, a catalytic domain, a 4-bladed β-propeller-like hemopexin domain and a transmembrane domain followed by a short cytoplasmic tail of only 20 amino acids [2]. Since its first appearance in the literature in the early 1990, MT1-MMP has been associated with cancer aggressiveness and its expression is widely accepted as a good prognostic indicator of poor survival [8,10,11,12,13].

The endogenous inhibitors of the MMPs, a group of structurally conserved proteins named Tissue Inhibitor of Metalloproteinases (TIMP-1 to -4), are relatively small molecules varying between 21–26 kDa. TIMPs inhibit the MMPs by inserting their wedge-like N-terminal domains, an area of the molecule termed “MMP-binding ridge,” into the catalytic clefts of the MMPs to form 1:1 tight-binding stoichiometric complexes that are essentially non-dissociable (examples include PDB #4ILW, #3V96). Despite sharing a similar tertiary configuration, the TIMPs differ vastly in their MMP selectivity. MT1-MMP, for instance, is sensitive to the inhibitory action of TIMP-2, -3 and -4 but not TIMP-1; the same is true for MMP-19 [14,15,16]. In an effort to delineate the molecular basis of TIMP-1’s selectivity, we have previously engineered a TIMP-1 variant named “T1^MT1^” that harbours a triple mutation “V4A/P6V/T98L” at the MMP-binding ridge. With a *K*_i_^app^ value of 1.66 nM against MT1-MMP, the affinity of T1^MT1^ was practically indistinguishable from that of the TIMP-2 (*K*_i_^app^ 1.30 nM), the natural inhibitor of MT1-MMP [14,17]. To further enhance the potency of T1^MT1^ against MT1-MMP, we couple the TIMP to the glycosyl-phosphatidyl inositol (GPI) anchor of the prion protein to create a membrane-tethered, high-affinity designer TIMP (named “T1^Pr αMT1^” hereafter) that is primarily expressed on the cell surface and co-localised with cellular MT1-MMP. Here, we show that not only could T1^Pr αMT1^ abolish the gelatin degrading ability of cellular MT1-MMP in HT1080 fibrosarcoma cells, the TIMP was also capable of preventing renal carcinoma cell (CaKi-1) tumorigenesis in in vitro and in vivo settings. Renal carcinoma is a highly metastatic and refractory cancer type in which MT1-MMP is known to be overexpressed [12,18,19,20]. Our unique approach of TIMP anchorage, we believe, may offer an opportunity for the development of novel therapeutics aiming at renal carcinoma treatment.

## 2. Results

### 2.1. “T1^Pr αMT1^”: A Membrane-Tethered, High-Affinity Designer TIMP-1 Tailored Specifically for MT1-MMP Inhibition

**Figure 1:**
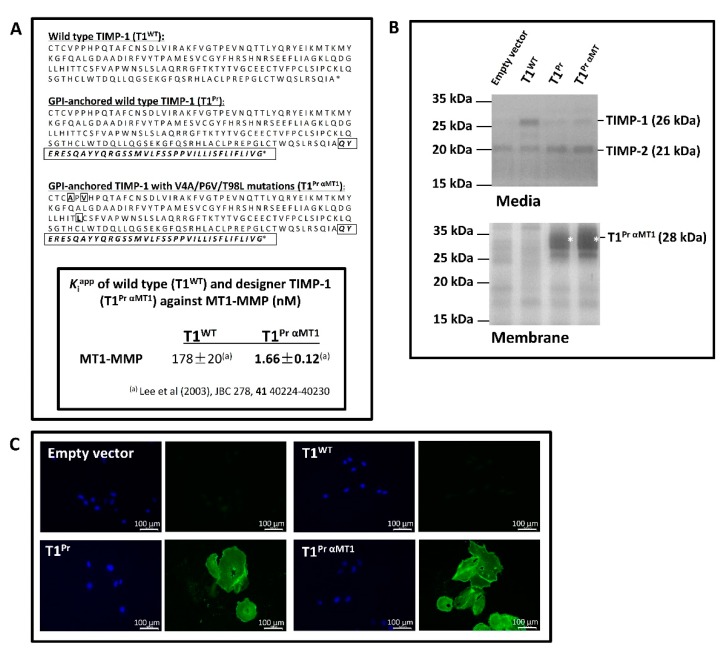
“T1^Pr αMT1^”: a GPI-anchored designer TIMP-1 tailored for MT1-MMP inhibition. (**A**) Top panel: Sequences of the wild type (T1^WT^) and designer TIMP-1s (T1^Pr^ and T1^Pr αMT1^) tailored for MT1-MMP inhibition. (1) T1^WT^: wild type TIMP-1 (2) T1^Pr^: GPI-anchored TIMP-1 (3) T1^Pr αMT1^: GPI-anchored “designer” TIMP-1 that carries a “V4A/P6V/T98L” triple mutation developed for MT1-MMP inhibition. Note that residues not native to TIMP-1 are boxed and highlighted in bold. Lower panel: inhibitory constant *K*_i_^app^ of the designer TIMP-1 “T1^Pr αMT1^” against MT1-MMP. In contrast to T1^WT^ which displayed a negligible affinity for MT1-MMP (*K*_i_^app^ = 178 nM), the mutant T1^Pr αMT1^ was a superb inhibitor of the proteinase (*K*_i_^app^ = 1.66 nM) [22]. (**B**) Sequestration of T1^Pr^ and T1^Pr αMT1^ to the cell membrane of the renal carcinoma cells CaKi-1 as revealed by reverse zymography. While T1^WT^ was secreted to the conditioned media, T1^Pr^ and T1^Pr αMT1^ were sequestered exclusively to the cell membrane (GPI-anchored TIMPs highlighted by asterisks *). (**C**) Non-permeabilised immunostaining with a TIMP-1 antibody confirmed successful immobilisation of T1^Pr^ and T1^Pr αMT1^ on CaKi-1 cell surface as visualised under a fluorescent microscope. The adjacent panels show the same cells stained with 4′,6-diamidino-2-phenylindole (DAPI).

Listed in Figure 1A are the amino acid sequences of all the TIMP-1 constructs in this study. Apart from the wild-type TIMP-1 (T1^WT^), we have created a GPI-anchored TIMP-1 (T1^Pr^) as well as a GPI-anchored “designer” TIMP-1 named “T1^Pr αMT1^” tailored specifically for MT1-MMP inhibition. With a *K*_i_^app^ value just over 170 nM, T1^WT^ is a known poor inhibitor of MT1-MMP. “T1^Pr αMT1^,” by contrast, is a GPI-anchored “designer” TIMP-1 that carries a “V4A/P6V/T98L” triple mutation tailored to fit the catalytic pockets of MT1-MMP [14,15]. As shown in the enclosed table, the affinity of the TIMP for MT1-MMP (*K*_i_^app^ 1.66 nM) is a vast improvement compared to that of the wild-type TIMP-1. Affinity aside, another distinctive feature of T1^Pr^ and T1^Pr αMT1^ noteworthy of interest is their C-terminal sequences “*QYERESQAYYQRGSSMVLFSSPPVILLISFLIFLIVG*” that bear a sharp resemblance to the signal peptide that encodes for human prion GPI anchor. Among the numerous GPI signal sequences in the database, we intentionally chose the prion protein as a targeting device due to the similarities in the way PrP^Sc^ (the scrapie form of prion) and MT1-MMP are localised at the membrane ruffles [21]. By navigating to the vicinity of MT1-MMP, we hope to maximise the effect of the TIMP at the site where its activity is most desired.

**Figure A1:**
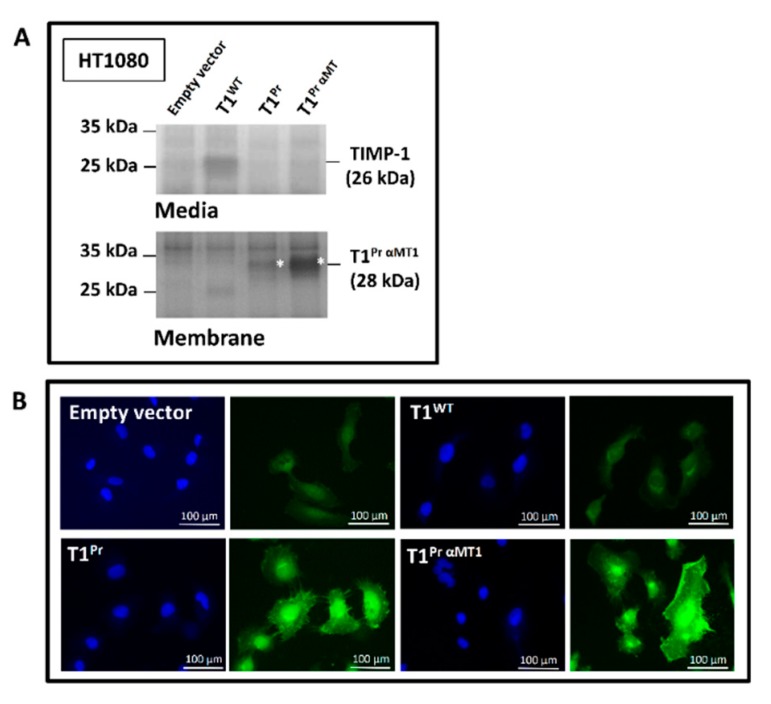
**Immobilisation of T1^Pr^ and T1^Pr αMT1^ on the membrane of fibrosarcoma cells HT1080.** (**A**) Sequestration of T1^Pr^ and T1^Pr αMT1^ to the membrane of HT1080 cells as shown by reverse zymography. (**B**) Non-permeabilised immunostaining with a TIMP-1 antibody confirmed immobilisation of T1^Pr^ and T1^Pr αMT1^ on HT1080 cell surface. The adjacent panels show the same cells stained with DAPI.

**Figure A2:**
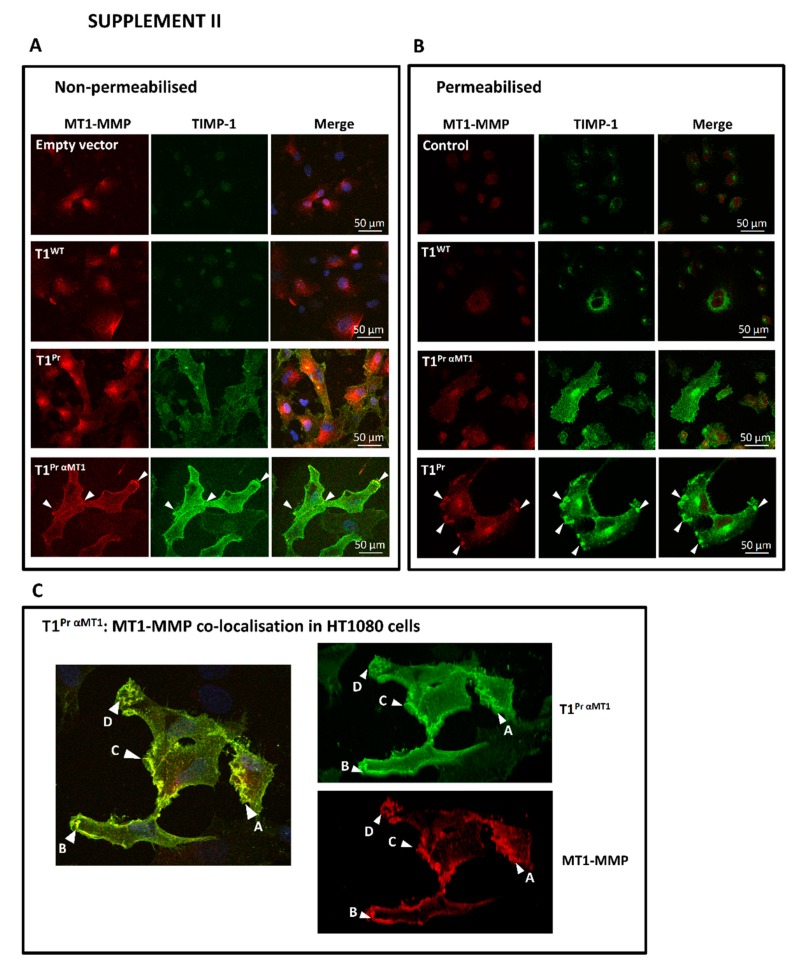
**Co-localisation of T1^Pr αMT1^ with MT1-MMP in stably transduced HT1080 cells.** Stably transduced HT1080 cells were co-immunostained with antibodies against TIMP-1 and MT1-MMP under non-permeabilising and permeabilising conditions. (**A**) Superb co-localisation of T1^Pr αMT1^ with MT1-MMP throughout the entire cell surface and membrane ruffles. (**B**) Permeabilised immunostaining showing intense T1^Pr αMT1^:MT1-MMP co-localisation at the membrane ruffles. (**C**) Orthogonal views highlighting co-localisation of T1^Pr αMT1^ with MT1-MMP on the cell surface and membrane ruffles (T1^Pr αMT1^:MT1-MMP co-localisation indicated by arrow heads).

Enclosed in Figure 1B and Figure A1 are reverse zymography gels illustrating the sequestration pattern of the TIMPs following stable transduction in the renal carcinoma cells CaKi-1 (Figure 1B) and fibrosarcoma cells HT1080 (Figure A2). While much of T1^WT^ (ca. 26 kDa) was secreted to the conditioned media, the GPI-anchored TIMP-1s T1^Pr^ and T1^Pr αMT1^ were detected exclusively in the membrane extracts (marked by asterisks *). There was no trace of the TIMPs in the conditioned media. Subsequent immunostaining carried out under non-permeabilised conditions confirmed that T1^Pr^ and T1^Pr αMT1^ had indeed been immobilised on the cell surface as well as the cell edges where membrane ruffling occurs (Figure 1C and Figure A2; images captured with a fluorescent microscope).

### 2.2. Localisation of T1^Pr αMT1^ on CaKi-1 Cell Surface and Membrane Ruffles

**Figure 2:**
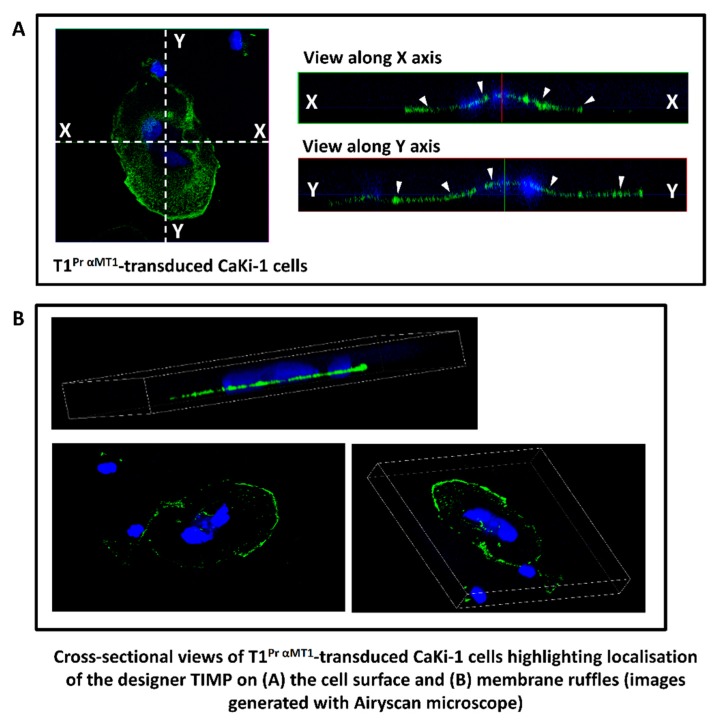
Cross-sectional views highlighting cellular localisation of T1^Pr αMT1^ in stably transduced CaKi-1 cells. CaKi-1 cells stably transduced with T1^Pr αMT1^ was immunostained with a TIMP-1 antibody under non-permeabilised conditions and visualised with an Airyscan confocal microscope. (**A**) Orthogonal views showing immobilisation of T1^Pr αMT1^ on the cell surface (indicated by arrowheads). (**B**) Concentration of T1^Pr αMT1^ at the cell edges as confirmed by 3D projection views of the membrane ruffles.

To analyse the distribution pattern of the GPI-TIMPs in greater detail, we compiled a 3D figure of a CaKi-1 cell cluster transduced with T1^Pr αMT1^ using cross-sectional images from confocal microscopy. Contained in Figure 2A are two orthogonal views (Y vertical; X horizontal) illustrating T1^Pr αMT1^’s coverage of almost the entire cell surface (positions of the TIMP are indicated by arrow heads). Figure 2B, on the other hand, highlights how the designer TIMP was concentrated at the cell edges where membrane ruffles are the thickest. The results confirmed that T1^Pr αMT1^ had indeed been targeted to the cell surface as intended.

### 2.3. Superb Co-Localisation of T1^Pr αMT1^ with Cellular MT1-MMP on the Cell Surface and Membrane Ruffles of CaKi-1 and HT1080 Cells

**Figure 3:**
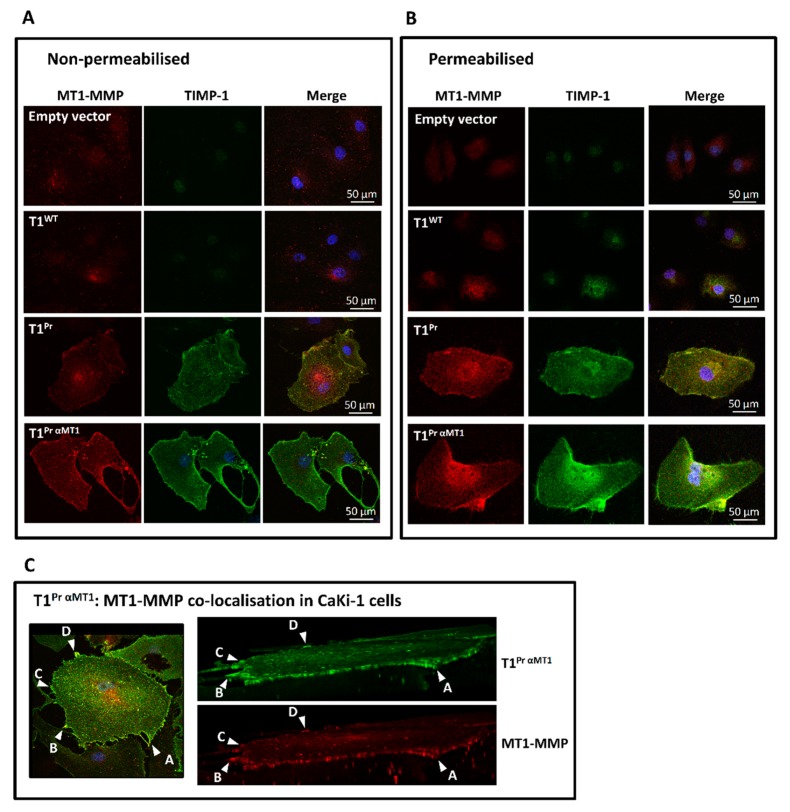
T1^Pr αMT1^:MT1-MMP co-localisation in stably transduced CaKi-1 cells. CaKi-1 cells stably transduced with the TIMPs were co-immunostained with antibodies against TIMP-1 and MT1-MMP under non-permeabilising and permeabilising conditions. (**A**) While the signal of T1^Pr^:MT1-MMP was weak and limited to the cell edges, T1^Pr αMT1^ col-localised with MT1-MMP intensely throughout the entire cell surface and much of the membrane ruffles. (**B**) Permeabilised immunostaining showing intense co-localisation of T1^Pr αMT1^ with MT1-MMP at the perinuclear regions as well as membrane ruffles. (**C**) Intense T1^Pr αMT1^:MT1-MMP co-localisation at the cell ruffles as highlighted by non-orthogonal (left) and orthogonal (right) views of the same CaKi-1 cell. Arrowheads A, B, C and D in the panels denote the same cell sites as viewed from a non-orthogonal (left) and orthogonal (right) perspectives.

Further immunostaining under non-permeabilising and permeabilising conditions showed that, not only could T1^Pr αMT1^ be detected in high abundance at the cell edges, it co-localised with MT1-MMP throughout almost the entire cell perimeters (Figure 3A for CaKi-1; Figure A1 for HT1080). Besides co-localisation on the cell membranes, the TIMP also showed unmistakable signs of co-localisation with MT1-MMP inside the cells. Figure 3B and Figure A2 are two collages of images that demonstrate the intracellular distribution patterns of T1^Pr αMT1^ under permeabilised staining conditions in the two cancer cell types. In comparison to T1^Pr αMT1^, T1^Pr^ could also be detected in sections of the membrane ruffles although the overall intensity was much weaker presumably due to its lack of affinity for MT1-MMP. In addition to Figure 3A,B (and Figure A1 and Figure A2), further evidence of T1^Pr αMT1^:MT1-MMP co-localisation is provided by the set of orthogonal images in Figure 3C (and Figure A2) taken under non-permeabilised conditions that highlight the whereabouts of T1^Pr αMT1^:MT1-MMP co-localisation at the membrane ruffles and cell surface.

### 2.4. T1^Pr αMT1^ Abolishes MT1-MMP-Mediated Gelatinolytic Capability of HT1080 in Cell-Based Setting

**Figure 4:**
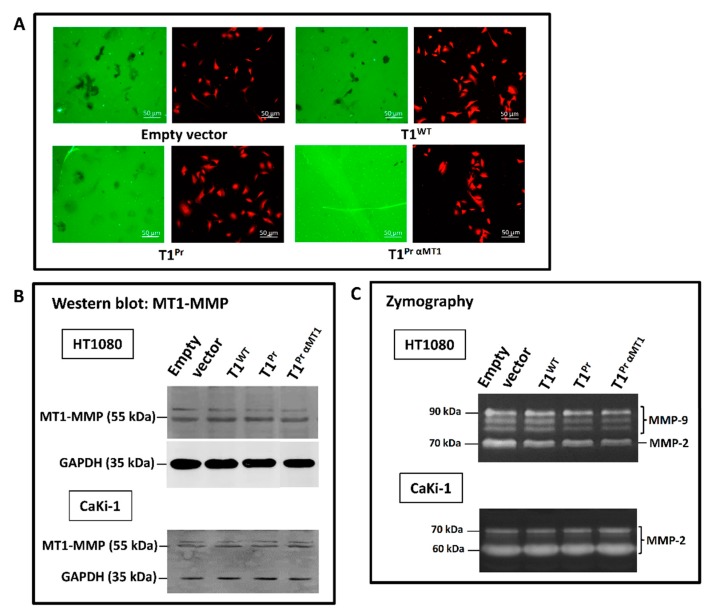
T1^Pr αMT1^ is a potent inhibitor of cellular MT1-MMP. (**A**) HT1080 fibrosarcoma cells stably transduced with the designer TIMPs were seeded on fluorescent gelatin-coated chamber slides overnight to allow gelatin degradation. In contrast to the empty vector-, T1^WT^- and T1^Pr^-expressing cells which left trails of smudgy, dark splotches of degraded gelatin on the chambers, T1^Pr αMT1^-transductants were completely devoid of gelatinolytic ability. The adjacent panels show the same cells stained with an anti-MT1-MMP antibody. (**B**) Despite its potency in gelatinolytic activity suppression, T1^Pr αMT1^ had no apparent effect on MT1-MMP expression as shown by immunoblotting of the cell lysates of stably transduced HT1080 and CaKi-1 cells. (**C**) T1^Pr αMT1^ expression has no impact on pro-MMP-2 and -9 (as in the case of HT1080) processing as revealed by gelatin zymography analysis of the conditioned media of HT1080 and CaKi-1 transductants.

To assess the inhibitory potency of T1^Pr αMT1^ against native MT1-MMP in a cell-based environment, we also transduced the TIMPs into the metastatic fibrosarcoma cells HT1080 which have been previously shown to exhibit a strong gelatin-degrading proficiency on immobilised fluorescent gelatin [22]. Though not an exclusive assay for MT1-MMP, the experiment should provide us with a clue as to the potency of the TIMPs against the protease under cellular setting. Figure 4A is a collection of images that succinctly summarise the relative potency of the various TIMPs against cellular MT1-MMP. In contrast to the large and occasionally smudgy splotches detected in the empty vector, T1^WT^ and T1^Pr^ chambers, there was almost no sign of gelatin degradation in the slide in which T1^Pr αMT1^-transductants occupied.

Further immunostaining and zymography analysis carried out on the cell lysates and conditioned media of stably transduced HT1080 and CaKi-1 cells indicated no change in MT1-MMP expression as well as pro-MMP-2 processing patterns as a result of the TIMPs’ expression (Figure 4B,C).

### 2.5. T1^Pr αMT1^ Expression Triggers Cellular Senescence in CaKi-1 Cells

**Figure 5:**
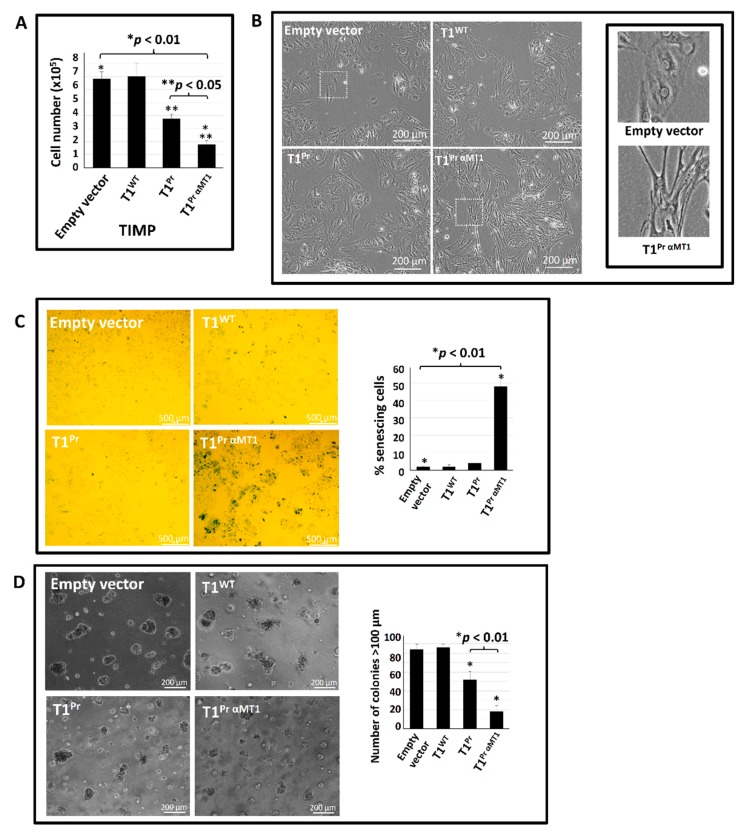
T1^Pr αMT1^ triggers cellular senescence in CaKi-1 cells as well as inhibits CaKi-1 proliferation in Matrigel suspension. (**A**) T1^Pr αMT1^-transduced CaKi-1 cells proliferated at a substantially slower rate than its T1^WT^ and T1^Pr^ counterparts under 2D culture conditions (* *p* < 0.05 vs. T1^Pr^). (**B**) In contrast to the typical phenotypic appearance of epithelial cells, T1^Pr αMT1^-transduced CaKi-1 cells appeared unusually large and “stretched” with no defined shape or boundary. Insets: close-up images highlighting the morphological difference between the empty vector- and T1^Pr αMT1^-transductants. (**C**) T1^Pr αMT1^ triggered an upsurge of SA-β-gal activity as demonstrated by X-gal staining (* *p* < 0.01 vs. other TIMPs). (**D**) While many of the empty vector-, T1^WT^- and T1^Pr^-transduced CaKi-1 cells developed into colonies > 100 μm within 25 days of incubation in Matrigel, T1^Pr αMT1^-transductants proliferated at a substantially slower rate (* *p* < 0.01 vs its runner up T1^Pr^). Results in the bar chart represent the average of three technical repeats ± S.E.M.

A distinctive feature of T1^Pr αMT1^-transduced CaKi-1 cells we noticed during the course of this study was their excessively slow proliferation rate in 2D culture conditions. Indeed, the typical proliferation rate of T1^Pr αMT1^-transductants was no higher than 26% of that of the empty vector and T1^WT^ controls (Figure 5A). Apart from a sluggish growth rate, the cells also appeared unusually stretched without a defined shape or boundary (Figure 5B; enlarged images in insets). Suspected to be signs of cellular senescence, we thus examined the level of senescence-associated β-galactosidase (SA-β-Gal) activity of the transductants using X-gal as a substrate [23]. Figure 5C shows that, whilst only a small proportion (2–4%) of the empty vector-, T1^WT^- and T1^Pr^-transduced cells developed blue colour upon incubation with X-gal, intense staining were detected in T1^Pr αMT1^-transductants. With an average percentage of blue cells approaching 50%, the incidence of senescing cells in T1^Pr αMT1^ population was far higher than those of the other TIMP transductants (* *p* < 0.01).

The effects of T1^Pr αMT1^ on cell proliferation is even more pronounced in the confine of Matrigel suspension. As shown in Figure 5D, without interference from the TIMPs, control CaKi-1 cells rapidly developed into colonies of irregular size in excess of 100 μm within 25 days of seeding. While the impact of T1^WT^ was minimal, T1^Pr^ appeared to exhibit an inhibitory effect though not by a large margin. T1^Pr αMT1^, in contrast, demonstrated an impressive anti-tumorigenesis efficacy as there were far fewer colonies that reached 100 μm by the end of the incubation period (*p < 0.01 vs. T1^Pr^).

### 2.6. T1^Pr αMT1^ Expression Causes Accumulation of Fibronectin, Collagen I and Laminin at the Pericellular Matrices

**Figure 6:**
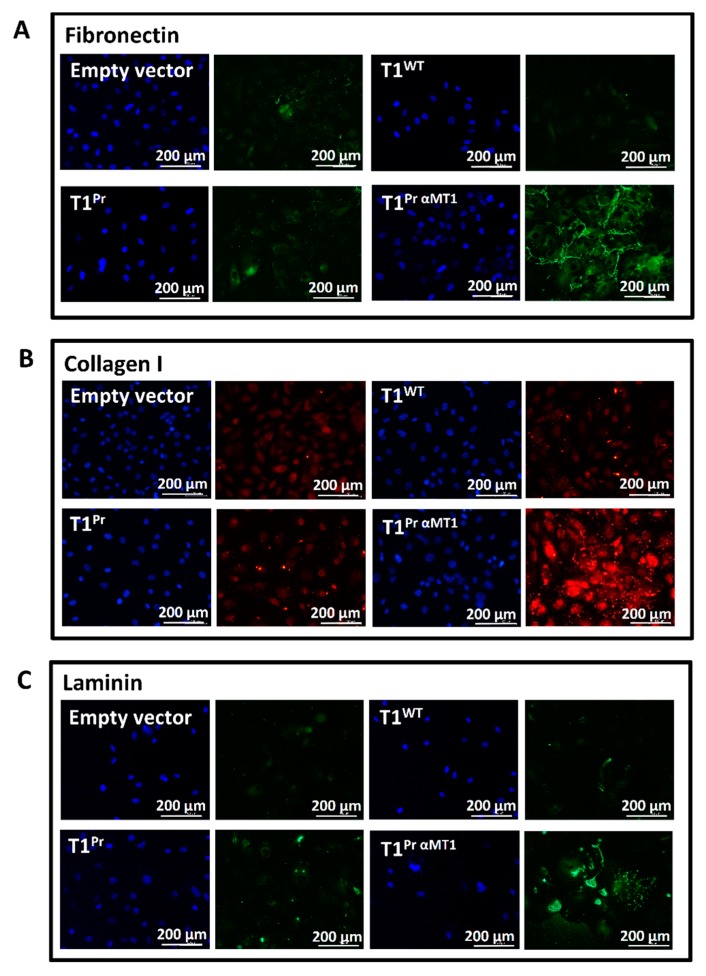
Pericellular accumulation of fibronectin, collagen I and laminin in T1^Pr αMT1^ transductants. Immunostaining showing accumulation of (**A**) Fibronectin (**B**) Collagen I and (**C**) laminin in T1^Pr αMT1^-transduced CaKi-1 cells. The adjacent panels show the same cells stained with DAPI.

Given the pivotal role MT1-MMP plays in ECM turnover and modulation, we were keen to find out if the uncharacteristic cell behaviours observed in Figure 5 were the outcomes of an altered cellular microenvironment. To this end, we stained the cells with a range of ECM antibodies and the results are summarised in Figure 6. In total contrast to the near-barren scenes observed in the empty vector-, T1^WT^- and T1^Pr^-transductants, there was a large amount of the macromolecules fibronectin, collagen I and laminin in the slides on which T1^Pr αMT1^-transductants were cultured. Another observation of interest in the figure is the rather dense and disorderly extracellular fibronectin/collagen I bundles that did not appear to follow an organised or recognisable pattern.

### 2.7. T1^Pr αMT1^ Inhibits CaKi-1 Growth in NOD/SCID Xenograft 

**Figure 7:**
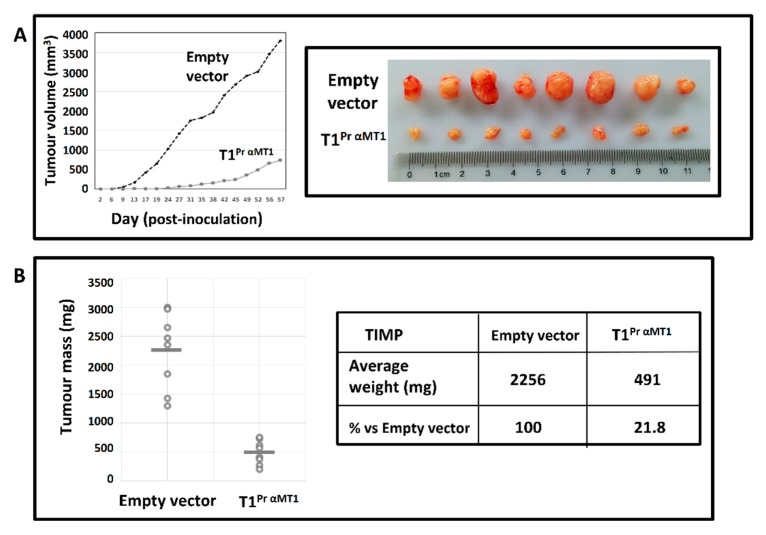
T1^Pr αMT1^ inhibits CaKi-1 proliferation in NOD/SCID mouse model. (**A**) Left: tumour growth curves for the control (empty vector) and T1^Pr αMT1^ implants over a 57-day period. Without the suppressive effects of the TIMPs, CaKi-1 cells rapidly developed into tumours within 10 days of inoculation in NOD/SCID mice. T1^Pr αMT1^-transduced cells, in contrast, only showed sign of tumour formation after 20 days of inoculation. Right panel: surgically removed control and T1^Pr αMT1^ tumours at the end of the experiment. (**B**) Scattered chart showing individual masses for the control (average 2256 mg) and T1^Pr αMT1^ (average 491 mg) tumours upon completion of the study (* *p* < 0.01). The mean for each group is represented by a grey bar.

Under in vivo conditions, the anti-proliferative effect of T1^Pr αMT1^ was equally, if not more impressive. Figure 7A is a summary of our findings on day-57 when the study reached a humane endpoint (*n* = 8). Without the suppressive effect of T1^Pr αMT1^, CaKi-1 tumours rapidly emerged in every control NOD/SCID mouse within 10 days of inoculation. Uninterrupted tumour growth was recorded in all the control mice from day-10 to -57 when the average tumour volume reached a very substantial 3,810 mm^3^. In contrast, T1^Pr αMT1^ tumours only started to appear after 20 days of inoculation. By the time the experiment was concluded on day-57, the average tumour volume for T1^Pr αMT1^ was no higher than 750 mm^3^, a fraction (20%) of that of the control group.

The individual masses for all the tumours following post-mortem surgery are shown in the scattered chart in Figure 7B. While the control tumours were all ranged within 1298–3005 mg (average = 2256 mg), the average mass for T1^Pr αMT1^ tumours was only 491 mg, a mere 22% of that of the control group (* *p* < 0.01).

## 3. Discussion

The findings in this study are significant for two reasons. First and foremost, our results have proven for the first time that the prion molecule, notwithstanding its infamy as an infectious agent, can be exploited as a targeting device in TIMP engineering. Collective evidence from microscopy, biochemical and cell-based assays, as we have shown here, are all unequivocally supportive of the use of the prion GPI as a means of targeting TIMP-1 to cellular MT1-MMP. The second significance of our findings lies in the demonstration of the anti-tumorigenesis efficacy of T1^Pr αMT1^ in the renal carcinoma cells CaKi-1. Renal carcinoma is a highly invasive and refractory cancer type for which there is no effective conventional therapy [18]. The invasive potential of the cancer, as shown by Petrella and co-workers, is linked to MT1-MMP not only through its function as a major matrix-degrading enzyme but also the mediating role it plays in the cleavage of adhesion molecules and other MMPs [12,20]. As such, there have long been calls for MT1-MMP to be listed as a prime target for therapeutic intervention in various types of invasive cancers including renal carcinoma [4,5,12].

Based on the data obtained, we can construct a rather straightforward rationale to explain the effectiveness of T1^Pr αMT1^ as seen in the animal study. As evident from the microscopy images in Figure 2, T1^Pr αMT1^ is expressed and segregated primarily at the membrane ruffles where MT1-MMP is mostly found. Given the TIMP’s in-built affinity for MT1-MMP (*K*_i_^app^ 1.66 nM; Figure 1), the inhibitor:enzyme interaction that ensues would inevitably lead to a downregulation in the proteolytic functions of MT1-MMP—a fact clearly reflected by the outcomes of the gelatinolytic assay in Figure 4. Inactivation of MT1-MMP, in turn, causes a disruption in ECM turnover as exemplified by evidence of fibronectin/collagen I/laminin accumulation in Figure 6. Maintenance of normal mechanical properties of the ECM is fundamental to cellular and tissue health (reviewed by Humphrey et al. [24]). A dramatic alteration in ECM homeostasis, as in the case of T1^Pr αMT1^, would likely bring about a disruption in normal “mechano-transduction” signalling between the nucleus and extracellular compartments. A direct consequence of a mechano-transduction failure, as reviewed by Humphrey et al., is a senescence response characterised by cell hypertrophy, a slowdown in proliferation and an upsurge in SA-β-gal activity as demonstrated in Figure 5 [24]. As a consequence of the T1^Pr αMT1^-transductants’ inability to modulate the extracellular microenvironment in order to undergo cytoskeletal reorganisation required for proliferation, the T1^Pr αMT1^ inoculums thus failed to adapt and prosper in the newly introduced in vivo environment [25].

## 4. Materials and Methods

### 4.1. Materials

All the reagents used in this study were supplied by ThermoScientific USA (Waltham, MA, USA) unless otherwise stated. Antibodies against TIMP-1 (Abcam: Ab1827) (Cambridge, MA, USA), MT1-MMP (Abcam: Ab38970), fibronectin (R&D Systems: MAB 19181) (Minneapolis, MN, USA), collagen I (Abcam: Ab34710) and laminin (Abcam: Ab11575) were purchased either from R&D Systems or Abcam, USA. Senescence detection kit and Matrigel^®^ were products from Biovision (San Francisco, CA, USA) and BD Biosciences (San Jose, CA, USA). HT1080 and CaKi-1 cell lines were acquired from the Shanghai Cell Repository, Chinese Academy of Science (Shanghai, China).

### 4.2. Cloning, Lentiviral Transduction, Reverse Zymography and Immunofluorescence Microscopy

The procedure for the creation of the soluble domain of T1^Pr αMT1^ by site-directed mutagenesis has been described in our previous paper [14]. The C-terminal prion tag was added by overlapping polymerase chain reaction (PCR) with three reverse primers bearing the following sequences (5′-GGCCTGAGATTCCCTCTCGTACTGGGCTATCTGGGACCGCAGGGACTG-3; 5′-CACAGGTGGGGAGGAGAAGAGGACCATGCTCGATCCTCTCTGGTAATAGGCCTGAGATTCCCTCTCGTACTG-3′ and 5′-TAAACGGGCCCTCATCCCACTATTAGGAAGATGAGGAAAGA GATCAGGAGGATCACAGGTGGGAGGAGAAGAGGAC-3′) with *Pwo* DNA polymerase. The PCR amplicon was digested with *EcoR* I and *Apa* I restriction enzymes before being subcloned into a pLVX-puro vector (Takara) for lentiviral packaging. All the clones generated in this study had been sequenced in both strands to confirm that no unwanted mutation had been introduced into the cDNAs during the PCR and cloning processes. The procedures for TIMP lentiviral transduction and reverse zymography were essentially identical to the ones elaborated previously [22]. For immunofluorescence, cells seeded in Nunc^®^ Lab-Tek II Chamber Slides^®^ were fixed in 4% paraformaldehyde prior to blocking with 5% bovine serum albumin in phosphate buffer saline (PBS). Following overnight incubation in primary antibodies at 4 °C, the cells were rinsed with PBS before further incubation in Alexa Fluor^®^ 488 (or 555)-conjugated anti-mouse and/or anti-rabbit secondary antibodies. Cell visualisation was performed with a Zeiss LSM880 Airyscan^®^ confocal microscope usually under 20× or 40× magnification.

### 4.3. Gelatin Degradation Assay

HT1080 cells transduced with the TIMPs (approximately 1000 cells) were seeded overnight in Nunc^®^ Lab-Tek II Chamber Slides^®^ pre-coated with 0.5 mg/mL porcine Oregon Green^®^ 488-conjugated fluorescent gelatin. Following fixation in paraformaldehyde, the slides were probed with an anti-MT1-MMP antibody before visualisation with a Nikon Eclipse Ni fluorescent microscope for degraded gelatin patches.

### 4.4. Cellular Senescence Assay with X-Gal

Caki-1 cells stably transduced with the TIMPs were cultured in a 12-well plate for at least 10 days before staining with X-gal as per manufacturer’s instructions. Following brief washing with PBS, the cells were examined under a Nikon Eclipse Ti inverted microscope for signs of blue precipitates. 

### 4.5. Renal Carcinoma Clonogenic Development in Matrigel

Clonogenic assay in Matrigel was carried out essentially as described [26]. The number of colonies > 100 μm after 25 days of incubation were counted and averaged for analysis. To ensure reliability, the assay had been performed in duplicate for at least three times.

### 4.6. Tumour Development Study in Non-Obese Diabetic/Severe Combined Immuno-Deficient (NOD/SCID) Model

All the experiments involving the use of animals were performed at GenePharma Co. Ltd. (animal licence registration number SYXK (Su) 2014-0054), Singapore Industrial Park, Biobay, Suzhou in strict accordance to the regulations outlined in the National Guidance for Animal Care, China. For growth evaluation, 4 × 10^6^ cells in 30% Matrigel/DMEM suspension were inoculated subcutaneously to the left or right flanks of 6-week old male NOD/SCID mice (*n* = 8 per group) to allow tumour development for 57 days. To calculate the tumour volume, the following formula was employed: *Volume* = *Length* × (*Width)*^2^ × π/6 where the width is defined as the smaller of the two perpendicular diameters. To ensure reproducibility, the experiment had been independently performed twice. This study does not require the use of human participants, data or tissues.

### 4.7. Statistical Analysis

Statistical analysis was carried out using the online calculator in the www.socscistatistics.com website. Statistical significance was determined by Student’s *t*-test usually under a two-tailed hypothesis.

## 5. Conclusions

In conclusion, we have shown that the GPI anchor of the prion molecule could be exploited as a molecular device for TIMP targeting. T1^Pr αMT1^, the GPI-anchored designer TIMP-1 created in this study, is a potent MT1-MMP inhibitor with the potential of being further developed into an experimental therapeutic for renal carcinoma treatment. We are currently testing the efficacy of the TIMP against a range of cancers using approaches including purified proteins as well as adenoviral delivery system. The results will be made public upon complete satisfaction of all the patenting criteria.

## Abbreviations

ECM	extracellular matrix
GPI	glycosyl-phosphatidyl inositol
MMP	matrix metalloproteinase
MT1-MMP	membrane type 1-matrix metalloproteinase
TIMP	tissue inhibitor of matrix metalloproteinase
